# Supplementary material for: Impact of focus of attention on aiming performance in the first-person shooter videogame Aim Lab
Source: PLoS One. 2023 Jul 25;18(7):e0288937. doi: 10.1371/journal.pone.0288937 (PMC10368276; doi:10.1371/journal.pone.0288937)
Supplement: Attachment — Submitted filename: Review PlosOne.docx [file pone.0288937.s002.docx]

In this study, the authors investigated the impact of FoA on aiming performance in FPS videogame players at different levels of expertise (low vs. high). The topic is relevant, and the research is rigorously conducted and reported. However, some minor issues are needed to be addressed before publication:

- The authors stated that the participants were divided into two groups based on the hours they self-reported having played with any FPS (200-1000 and >1000). However, no other information further characterizing the study cohort has been reported. If possible, please report whether they were professional players, the mean and standard deviation of hours played overall and per day, and what kind of at-home strategy (internal vs. external FoA) they usually adopt.
- The formula used by the authors for the post-manipulation check is unclear. It would be better for the readers to add examples of participants meeting or non-meeting the criteria to be included in the statistical analysis.
- As the authors stated in the methods section, participants were asked to hold down the left-click mouse button to fire repeatedly during the task, which contrasts with the clicking-oriented aiming approach used in the vast majority of the FPS. This represents the most “ecological” shooting approach for the players, and have asked them to use a different strategy might have influenced their performance and adherence to task instruction. Please briefly address this topic also in the discussion section.
- Relative to the experimental procedure, the authors stated that experimental blocks were randomized and counterbalanced. This refers to the order of the two conditions (internal vs. external FoA) across participants. Please also clarify whether or not the targets’ size and speed during the trials were counterbalanced across conditions and participants.
- Fig. 1 caption, line 239: (>1001 hours) instead of (>1000 hours). Please correct.
